# BIMAM—a tool for imputing variables missing across datasets using a Bayesian imputation and analysis model

**DOI:** 10.1093/ije/dyab177

**Published:** 2021-09-06

**Authors:** Fadlalla G Elfadaly, Alex Adamson, Jaymini Patel, Laura Potts, James Potts, Marta Blangiardo, John Thompson, Cosetta Minelli

**Affiliations:** 1 School of Mathematics and Statistics, The Open University, Milton Keynes, UK; 2 National Heart and Lung Institute, Imperial College London, London, UK; 3 Department of Biostatistics & Health Informatics, Institute of Psychiatry, Psychology & Neuroscience, King’s College London, London, UK; 4 MRC Centre for Environment and Health, School of Public Health, Imperial College London, London, UK; 5 Department of Health Sciences, University of Leicester, Leicester, UK

**Keywords:** Multiple imputation methods, systematically missing data, Bayesian methods, Bayesian hierarchical models, R Shiny application

## Abstract

**Motivation:**

Combination of multiple datasets is routine in modern epidemiology. However, studies may have measured different sets of variables; this is often inefficiently dealt with by excluding studies or dropping variables. Multilevel multiple imputation methods to impute these ‘systematically’ missing data (as opposed to ‘sporadically’ missing data within a study) are available, but problems may arise when many random effects are needed to allow for heterogeneity across studies. We show that the Bayesian IMputation and Analysis Model (BIMAM) implemented in our tool works well in this situation.

**General features:**

BIMAM performs imputation and analysis simultaneously. It imputes both binary and continuous systematically and sporadically missing data, and analyses binary and continuous outcomes. BIMAM is a user-friendly, freely available tool that does not require knowledge of Bayesian methods. BIMAM is an R Shiny application. It is downloadable to a local machine and it automatically installs the required freely available packages (R packages, including R2MultiBUGS and MultiBUGS).

**Availability:**

BIMAM is available at [www.alecstudy.org/bimam].

**Key Features**

- When information is combined across datasets, multilevel multiple imputation of variables missing in some datasets should always be considered if a missing at random assumption is reasonable.
- Available approaches may not work well if there is heterogeneity and, to allow for that, too many random effects are required relative to the number of datasets.
- In this case, the performance of available imputation methods substantially improves when followed by a Bayesian analysis model.
- BIMAM implements a Bayesian joint imputation and analysis approach that works very well also in the presence of heterogeneity, which it fully allows for.
- BIMAM is a stand-alone online tool that is user-friendly and can be used by researchers not familiar with Bayesian methods.

## Introduction

In collaborative epidemiological projects that combine information across multiple datasets to estimate the associations of risk factors with a disease trait or find its best set of predictors, a major issue is how to deal with studies that have measured different sets of variables. This problem is referred to as systematically missing data, as opposed to sporadically missing data where values are missing for individuals within a dataset.

Following an approach similar to the widely used Multiple Imputation by Chained Equations (MICE) for sporadically missing data,^1^ methods to impute missing variables in a dataset based on information from the other datasets have recently been developed. Under a missing at random assumption, MICE imputes sporadically missing data through random draws from the posterior distribution of the missing values given the observed data, using a sequence of conditional regressions (linear models for continuous missing data, logistic for binary data, etc.). Multiple imputed datasets, created to reflect uncertainty in the imputation, are analysed separately and their results combined using Rubin's rules.^2^ Multilevel extensions of MICE to account for the non-independence of observations when combining datasets (clusters) have been developed using classical and Bayesian methods, some of which allow simultaneous imputation of both systematically and sporadically missing data.^3–6^ Whereas these methods are based on fully conditional specification (FCS) of the imputation model, where a conditional distribution is defined for each missing variable, others have been developed based on joint modelling (JM), where a multivariate joint distribution is specified for all variables in the imputation model.^7^ All these methods have been recently compared, modified and implemented in a single R package, *micemd*, by Audigier *et al.*^8^ They all generate multiple imputed datasets with results combined using Rubin’s rule, and the analysis of interest is typically performed within a classical framework.^8^

When pooling data from different populations, or from studies with different methods, there is often heterogeneity across datasets in the size of the association of both the risk factors with the outcome (analysis model), and the predictors with the missing variable (imputation model). This is what we observe in the project that has motivated our work, the Ageing Lungs in European Cohorts (ALEC) international study, which combines multiple datasets to identify risk factors for poor lung function and chronic obstructive pulmonary disease [www.alecstudy.org]. All imputation methods compared by Audigier *et al*.^8^ can accommodate heterogeneity, through specification of a random effect or a hierarchical distribution if using a Bayesian approach. In practice, however, they may not be able to provide accurate results if there are not enough data to estimate such heterogeneity, for example when there are too many random effects relative to the number of datasets.^4^^,^^6^ Bayesian methods tend to perform better than classical methods in this situation, but such advantage may be limited if the Bayesian framework is only used for the imputation and not for the analysis model, such as in the Bayesian imputation approaches reviewed by Audigier *et al.*^8^

In 2009, Jackson *et al*.^9^ proposed an integrated Bayesian approach where the imputation of systematically and sporadically missing data is performed jointly with the analysis of interest, and uncertainty in the imputation is fully accounted for without the need to create multiple imputed datasets. This method has been rarely used in practice, likely due to the required knowledge of Bayesian methods and the lack of a package implementing it. Here we present a user-friendly freely available tool, BIMAM—Bayesian IMputation and Analysis Model—that makes this approach accessible to researchers unfamiliar with Bayesian statistics.

We illustrate BIMAM using data from the European Community Respiratory Health Survey (ECRHS),^10^ a multicentre study part of the ALEC project. We empirically demonstrate BIMAM performance by artificially dropping variables in some centres and comparing the results after imputation with the results of the analysis of the original complete data (‘gold-standard’). We also compare BIMAM with two other imputation approaches, selected following the recommendations in Audigier *et al*.^8^ based on number and size of the clusters: the classical FCS-2stage method by Resche-Rigon and White^5^ for the imputation of both continuous and binary variables, and a combination of the FCS-2stage method and the Bayesian imputation method by Quartagno *et al*.^7^ for the imputation of continuous and binary variables, respectively.

## Implementation

### BIMAM model

The Bayesian approach by Jackson *et al*.,^9^ with imputation and analysis of interest fitted jointly, was originally described in a scenario of a binary outcome analysed using two datasets, with two categorical risk factors missing in one of them. We generalized it to: analyse both continuous and binary outcomes; impute any number of binary or continuous variables missing across any number of datasets; account for heterogeneity for all variables in both imputation and analysis models. The multilevel structure of the data is reflected in a hierarchical formulation of the imputation and analysis models, and sporadically and systematically missing data are imputed simultaneously.

The parameters of interest are estimated by an iterative Markov Chain Monte Carlo (MCMC) process based on Gibbs sampling, using the MultiBUGS package.^11^ Binary variables are imputed as latent normal variables using a probit link, and all variables (binary and continuous) are imputed jointly using a multivariate normal distribution. Non-informative prior distributions are used for all parameters. Details on the approach are reported in the Supplementary material, available as Supplementary data at *IJE* online.

Estimates for the parameters of interest in the analysis model correspond to the mean (or median) of the posterior distribution of the parameter, and 95% credibility intervals (95% CrI), the Bayesian analogous to the classical 95% confidence intervals (95% CI), correspond to the 2.5th and 97.5th percentiles of such distribution.

### BIMAM tool

BIMAM is a stand-alone user-friendly tool with instructions provided at each step, and is freely available at [www.alecstudy.org/bimam]. Screenshots of the tool are shown on page 13 of the Supplementary material. After uploading the dataset, a summary is provided which identifies variables with missing data. Using a drop-down list, the user is asked to specify: (i) the clustering variable (e.g. centre, study); (ii) outcome and covariates for both imputation and analysis models; (iii) for each covariate in both models, whether it is modelled as fixed or random effect. To run the MCMC analysis, the user also needs to specify: (a) length of ‘burn-in’ (initial iterations that are discarded to avoid any influence of the initial values on the results); (b) number of ‘updates’ (iterations used in the analysis—the larger the updates, the more accurate the results); (c) number of chains (number of separate MCMCs, used to assess model convergence). Initial values for all parameters are assigned by the tool (see page 3 of the Supplementary material). To speed convergence, BIMAM standardizes all variables (binary and continuous covariates and outcome) in both imputation and analysis models, with the exception of binary missing variables that are imputed as zeros and ones; all the regression coefficients of standardized covariates are then automatically ‘unstandardized’ back (details reported on page 4 of the Supplementary material). Together with the results, the tool shows the model running time as well as warning messages with recommendations: if the accuracy of the results is too low, → suggestion to increase number of updates if MCMC error (simulation error) >5% of the standard error of any parameter of interest; if convergence is not reached, → suggestion to increase burn-in period if Gelman-Rubin statistic (R-hat) >1.1.^12^ For users not familiar with Bayesian methods, meaning and implications of MCMC settings, MCMC error and R-hat are very briefly explained in pop-up windows next to the related field and in more detail in the online BIMAM manual. The results output presents the beta coefficients of all variables in the analysis model in a downloadable table, with posterior estimate, standard error and 95% CrI as well as MCMC error and R-hat. Advanced users can view and save the diagnostic plots (trace and density plots), and save the CODA files.

## Use

### BIMAM implementation and comparison with other approaches using ECRHS data

Using ECRHS data, we consider a linear regression model estimating the association of smoking (ever vs never), weight (kg), sex (female vs male), height (cm) and age (years) with a spirometric measure of airway obstruction, the ratio of forced expiratory volume in 1 s over forced vital capacity (FEV_1_/FVC, expressed as %). Starting with complete data from a sample of 6613 subjects from 10 ECRHS centres of size between 521 and 1047 (Supplementary Table, available as Supplementary data at *IJE* online), we artificially dropped weight (continuous) from one centre, smoking status (binary) from another, and both variables from three centres. Each missing variable was then imputed using a model that included all other covariates except for the other missing variable, as well as the outcome. Since peculiarities in the centres with missing variables may influence the results, to exclude the play of chance we considered 20 different scenarios, created from the complete data by randomly changing the five centres with dropped variables. Overall estimates for the beta coefficient of all risk factors in the analysis model were then obtained by averaging over the 20 different scenarios using the Rubin’s rule.

On the 20 scenarios, we also applied:

- same Bayesian hierarchical model as BIMAM analysis model on the complete data (gold standard), using MultiBUGS;
- same as above, but after dropping centres with missing variables (naïve analysis);
- classical FCS-2stage method for imputation of both continuous (weight) and binary (smoking) variables. This method, based on the two-stage estimator described for IPD meta-analysis,^13^ was proposed by Resche-Rigon and White^5^ and modified in Audigier *et al*.^8^; we implemented it using the 2l.stage commands of the *micemd* R package [https://cran.r-project.org/web/packages/micemd/micemd.pdf];
- FCS-2stage method for imputation of weight (continuous) and the Bayesian JM imputation method based on conjugate prior distributions by Quartagno *et al*.^7^ for imputation of smoking (binary), again using the *micemd* R package.

For both the FCS-2stage and the FCS-2stage/jomo approaches, we generated 20 imputed datasets, the results of which were combined using Rubin’s rules and compared with those of the gold standard, naïve analysis and BIMAM. For both approaches, a classical multilevel model (*lmer* R package) was used for the analysis model.

For all methods, we allowed for between-centre heterogeneity in all variables of the imputation and analysis models, since heterogeneity is expected in the international ECRHS study.

All analyses were performed in R version 3.3.2, including the Bayesian analyses for jomo (using the *micemd* package) and for BIMAM (using *R2MultiBUGS* to run MultiBUGS). For the Bayesian analyses, the length of burn-in was increased as needed to achieve convergence, assessed using R-hat (<1.1) as well as visual inspection of trace, autocorrelation and density plots; the number of updates was decided based on MCMC error (<5%). Further details and BUGS code for BIMAM are reported in the Supplement, available at *IJE* online.

**Figure 1:**
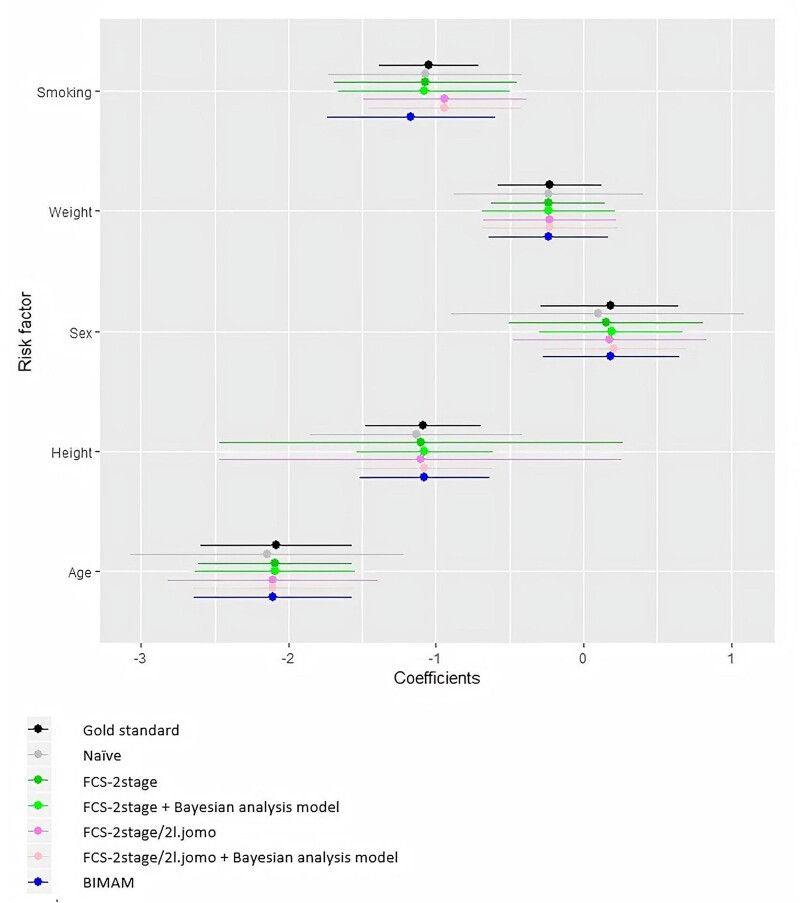
Beta coefficient and 95% CI or 95% CrI for the association of all risk factors with FEV1/FVC averaged over the 20 scenarios, for all methods. Coefficients for weight, height and age were multiplied by 10 (referring to increase of 10 kg, 10 cm and 10 years, respectively). CI: confidence interval; CrI: credibility interval; FEV1/FVC: ratio of forced expiratory volume in 1 s over forced vital capacity (expressed as %); FCS: fully conditional specification; BIMAM: Bayesian IMputation and Analysis Model

**Figure 2:**
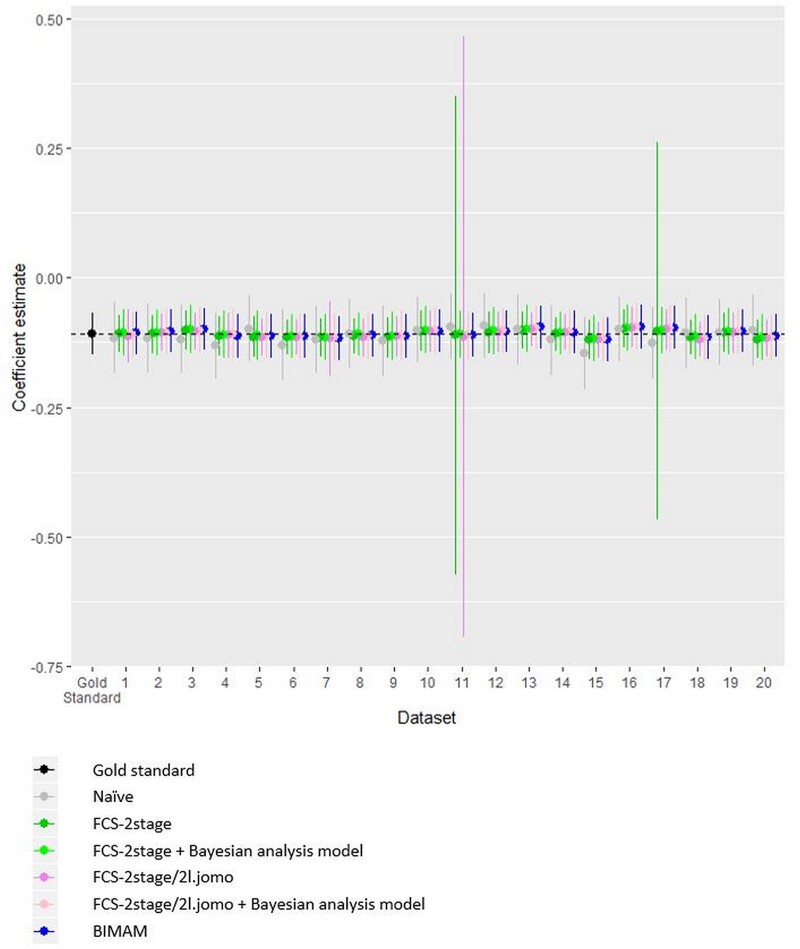
Beta coefficient and 95% CI or 95% CrI for the association of height with FEV1/FVC across the 20 scenarios, for all methods. The coefficient for height was multiplied by 10 (referring to increase of 10 cm). CI: confidence interval; CrI: credibility interval; FEV1/FVC: ratio of forced expiratory volume in 1 s over forced vital capacity (expressed as %); FCS: fully conditional specification; BIMAM: Bayesian IMputation and Analysis Model

Results for the association of the risk factors with FEV_1_/FVC, averaged over the 20 scenarios, are graphically compared across methods in Figure 1. Compared with the gold standard, point estimates showed little evidence of bias for any of the methods, but the methods differed in terms of precision. BIMAM performed better than the naïve analysis, with substantially narrower 95% CrI for all variables except smoking. FCS-2stage and FCS-2stage/jomo performed similarly to BIMAM, except for the effect estimate of height, where the two methods gave much wider confidence intervals. To a much lesser extent, this was also found for sex and age. Visual inspection of the results across the 20 scenarios for height showed that this was due to very unstable estimates in a couple of scenarios, in contrast with the stability of the results for BIMAM. This is shown for height in Figure 2 and for the other variables in the Supplementary Figure, available as Supplementary data at *IJE* online. Interestingly, by applying the FCS-2stage and FCS-2stage/jomo imputation approaches followed by the same Bayesian analysis model used in BIMAM, as opposed to the classical multilevel analysis used in the *micemd* package, we found that the advantage of BIMAM was largely explained by the Bayesian hierarchical framework used for the analysis model (Figure 1); this was confirmed by the higher stability of results across scenarios (Figure 2; Supplementary Figure, available as Supplementary data at *IJE* online).

The computational time for BIMAM, which was much reduced by using MultiBUGS as opposed to OpenBUGS and by standardizing all variables, was in line with the other methods: 11 min against 3 min for FCS-2stage and 20 min for the FCS-2stage/jomo.

## Conclusions

With the aim of increasing statistical power, modern epidemiology is moving from the analysis of single datasets by individual research groups to collaborative work with combined analysis of multiple datasets. The missingness of variables of interest in some of the datasets is commonly dealt with by either excluding such variables, thus impairing the performance of the final model, or excluding studies that have not measured them, thus reducing sample size and power. If a missing at random assumption is reasonable, imputation of missing variables across datasets should always be considered, and packages to implement multilevel methods to do this are available. However, problems may arise when fully allowing for heterogeneity in both imputation and analysis models if the number of datasets is small relative to the number of random effects required. This is the case in our example, where we show some instability of the results from the two imputation methods recommended based on number and size of clusters,^8^ the classical FCS-2stage and a combination of the FCS-2stage with the Bayesian jomo (for imputation of continuous and binary variables, respectively), which might result in wide confidence intervals for the coefficients of the analysis model. The Bayesian approach implemented in BIMAM, where imputation and analysis models are performed jointly, outperformed them and showed higher stability of the results. The problem of instability in the results from FCS-2stage and FCS-2stage/jomo approaches seemed to be largely solved by using a Bayesian hierarchical model instead of a classical multilevel model for the analysis of interest. This, however, is not implemented in the R packages available for these two approaches and requires experience with Bayesian methods. On the contrary, BIMAM is a user-friendly tool that does not require familiarity with Bayesian statistics and works very well when fully allowing for heterogeneity across a relatively small number of datasets.

## Supplementary Data

Supplementary data are available at *IJE* online

## Ethics approval

The ECRHS study, which is the source of the example dataset used in this paper, was performed with the approval of the corresponding local/regional committees for all participating centres, and with written informed consent obtained from all participants.

## Funding

This project has received funding from the European Union’s Horizon 2020 research and innovation programme under grant agreement No 633212.

## Data availability

The ECRHS dataset used to illustrate the application of BIMAM in this paper, and used as an example in the online manual, is available in the BIMAM tool at [www.alecstudy.org/bimam].

## Author Contributions

F.E. contributed to the design of the study, analysis and interpretation of data, development of the online tool and drafting of the paper; A.A. and J.Pa. contributed to the analysis and interpretation of data and testing of the online tool; L.P. contributed to the analysis and interpretation of data; J.Po. contributed to the acquisition of data and testing of the online tool; M.B. contributed to the design of the study and interpretation of data; J.T. contributed to the concept and design of the study and interpretation of data; C.M. contributed to the concept and design of the study, interpretation of data and drafting of the paper. All the authors critically revised the paper and approved the final version.

## Conflict of Interest

None declared.

## Supplementary Material

dyab177_Supplementary_Data
