# Supplementary material for: BIMAM—a tool for imputing variables missing across datasets using a Bayesian imputation and analysis model
Source: Int J Epidemiol. 2021 Sep 6;50(5):1419–25. doi: 10.1093/ije/dyab177 (PMC8580266; doi:10.1093/ije/dyab177)
Supplement: dyab177_Supplementary_Data [file dyab177_supplementary_data.docx]

**Supplementary Material**

In this Supplement, we provide a description of the original Bayesian approach proposed by Jackson et al. [1], together with the BUGS code used to implement it. We also include our R scripts to implement the other methods using the R package *micemd*, by Audigier et al. [2]. BIMAM and its documentation, including a manual with instruction for use, is available at [www.alecstudy.org/bimam](http://www.alecstudy.org/bimam).

Contents

[Details of the Bayesian approach 2](#_Toc69821683)

[Bayesian model implemented in BIMAM 3](#_Toc69821684)

[Supplementary Table 5](#_Toc69821685)

[Supplementary Figure 6](#_Toc69821686)

[BUGS code 9](#_Toc69821687)

[BIMAM online tool 13](#_Toc69821688)

[References 17](#_Toc69821689)

# Details of the Bayesian approach

Similarly to the other approaches for the imputation of systematically missing variables, the Bayesian method proposed by Jackson et al. [1] in 2009 can be used to impute a missing explanatory variable from one dataset based on at least one other dataset where this variable is fully observed. The idea is to use some other variables that are available in both datasets to build an imputation model for the missing variable based on the complete dataset(s). This imputation model is then used to predict the missing variable in the incomplete dataset. Jackson’s Bayesian approach jointly performs the imputation of systematically missing variables, as well as sporadically missing data, and the analysis of interest, with uncertainty in the imputation fully accounted for without the need to create multiple imputed datasets.

The approach was originally described in a scenario of a binary outcome, low birth weight, analysed using two datasets: an epidemiological study with complete data, and a large administrative dataset with two missing categorical variables, smoking (binary) and ethnicity (four categories). The two missing variables were combined in a single categorical variable with eight categories and imputed in the administrative dataset using all the variables in the analysis model, including the outcome, together with additional predictors of the missing variables used to improve the imputation. By adjusting the imputation model for the variables thought to govern selection into the epidemiological study, they assumed a missing-at-random mechanism for the missing variables in the register, since missingness is equivalent to inclusion in the portion of the data coming from the register rather than the study.

Jackson et al. proposed two approaches to implement their method, which differ in the way the information on the outcome is used for the imputation of the missing variables. The first approach consists of a joint imputation and analysis model (“full probability model”), where the outcome implicitly influences the prediction of the missing variables and is therefore not explicitly included in the imputation model. This approach can be computationally burdensome, and the authors proposed a second approach, where the process is divided into two stages. In the first stage, a Bayesian imputation model is implemented using the complete dataset; the posterior distributions of its coefficients are then used as prior distributions on the same model applied to the incomplete dataset to obtain a posterior distribution of the missing variable. In the second stage, the posterior distribution of the missing variable from the imputation model is then used as a prior distribution for the missing variable in the analysis model. In this approach the outcome is used as an explicit predictor of the missing variable in the imputation model in the first stage, and in the second stage a “cut function” is used to break the dependence of the outcome on the missing variable, so that outcome data are not used twice.

Using a slightly modified version of the second approach proposed by Jackson et at., with the cut function used to avoid a double use of the outcome data in a joint imputation and analysis model, we have generalised Jackson’s model to: analyse both continuous and binary outcomes; impute any number of binary or continuous variables missing across any number of datasets; account for heterogeneity for all variables in both imputation and analysis models. The multilevel structure of the data is reflected in a hierarchical formulation of the imputation and analysis models, and sporadically and systematically missing data are imputed simultaneously. Binary variables are imputed using latent normal variables and a probit link, and all variables (binary and continuous) are imputed jointly using a multivariate normal distribution. Non-informative prior distributions are used for all parameters. The parameters of interest are estimated by an iterative Markov Chain Monte Carlo (MCMC) process based on Gibbs sampling, using the MultiBUGS package [3].

For simplicity, the Bayesian model described in this section, with its BUGS code given on p. 9, is formulated slightly differently from the model implemented in BIMAM, where the code is generalised to reflect the user’s input and convergence is sped up by standardising all variables (see next section “Bayesian model implemented in BIMAM”). The results from the two formulations of the model are the same.

We used vague prior distributions for all parameters; for mean parameters we used a Normal (0, 1.0E-6), and for precision parameters we used a Gamma (1, 0.005). For the variance-covariance matrix of the bivariate normal distribution for the two systematically missing variables, weight (continuous) and smoking (binary – imputed using a latent normal variable), we have used the parameterization proposed by Wei and Higgins [4]. Using Cholesky decomposition, the method separates the variance–covariance matrix so that variances and correlations can be modelled separately, which makes it very flexible by allowing independent prior distributions to be placed on standard deviations and correlations. The method ensures that the matrix is positive-semidefinite, and we can then impose constraints on any of the variances, such as fixing the latent variable variance to 1. The variance-covariance matrix, sigma, is written as sigma=${V^{1/2}RV}^{1/2}$, where $V^{1/2}$ is a diagonal matrix with standard deviations on the main diagonal, and R is the correlation matrix. Cholesky decomposition is used to factor$R$ as$R=L^{T}L$, where$L$ is an upper-triangular matrix, with$L_{21}=0$:

$R=L^{T}L=\left( \begin{matrix} L_{11} & 0 \\ L_{12} & L_{22} \end{matrix} \right)\left( \begin{matrix} L_{11} & L_{12} \\ 0 & L_{22} \end{matrix} \right)$.

By putting$L_{11}=1$,$L_{12}\sim$Uniform (-1,1) and$L_{22}=\sqrt{1-L_{12}^{2}}$, the correlation matrix is parametrized as:

$R=\left( \begin{matrix} L_{11}^{2} & L_{11}L_{12} \\ L_{11}L_{12} & L_{12}^{2}+L_{22}^{2} \end{matrix} \right)=\left( \begin{matrix} 1 & Uniform(-1,1) \\ Uniform(-1,1) & 1 \end{matrix} \right)$.

The model used for our example was run with two chains, using a burn-in of 20,000 and 20,000 iterations. Convergence was assessed visually through examining trace plots, Brooks-Gelman-Rubin plots and the R-hat statistics, and found satisfactory (R-hat<1.1).

## Bayesian model implemented in BIMAM

The Bayesian model implemented in BIMAM is parameterised in the same way as above, but the code, which is generated based on the user’s input (see BIMAM online tool) is generalised to impute any number of missing variables across clusters (studies/centres) as well as sporadically missing data. For the variance-covariance matrix of the multivariate normal distribution, BIMAM uses simple formulas described in Wei and Higgins [4] for a general L matrix of any size to express its non-zero elements in terms of variables with Uniform (-1,1) prior distributions. As above, vague Normal (0, 1.0E-6) priors are used for all mean parameters, and vague Gamma (1, 0.005) priors for all precision parameters. Initial values of all mean parameters are randomly generated from Normal (0, 1) and those for precision parameters are randomly generated from Gamma (1, 1). Initials of the missing values of each continuous variable are randomly generated from a normal distribution parametrized with the sample mean of the observed values of that variable and their standard deviation. Initials for the missing values of each binary variables are randomly generated from Bernoulli (0.5) and the initials of their corresponding latent variables are generated from Normal (0, 1). Differently from the model described on p. 9, in order to speed convergence BIMAM standardises all variables (binary and continuous covariates and outcome) in both imputation and analysis models, with exception of binary missing variables that are imputed as zeros and ones. All the regression coefficients of standardised covariates are then automatically “unstandardised” back, as explained in the Box below.

**BOX: “Unstandardisation” of the regression coefficients of standardised covariates**


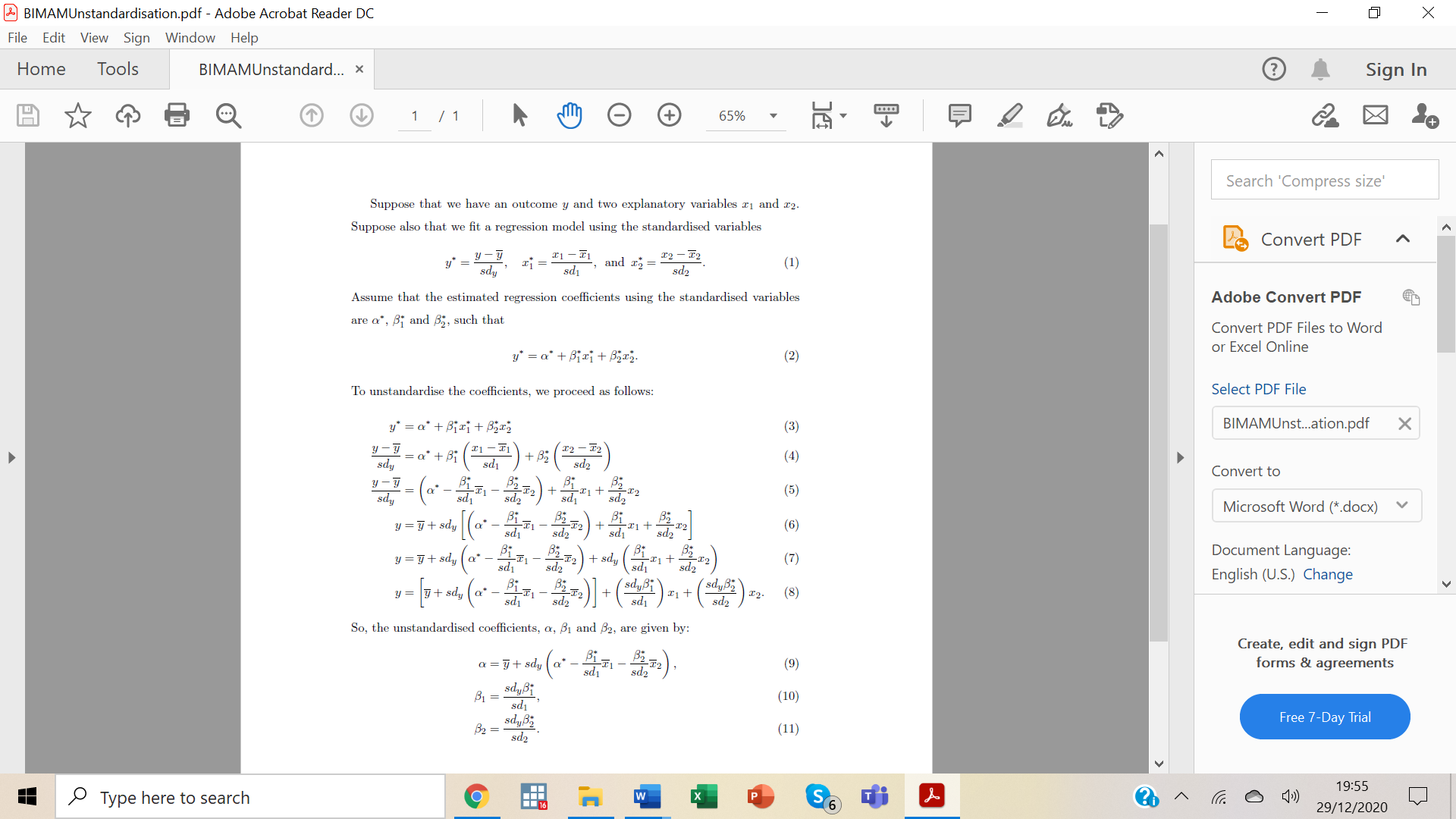


# Supplementary Table

Characteristics of the study population in the ECRHS 10 centres used as illustrative example.

| City/country | N | FEV1/FVC (%) Mean (SD) | Ever smoked (%) | Age (years) Mean (SD) | Height (cm) Mean (SD) | Weight (kg) Mean (SD) | Sex (Male)  (%) |
| --- | --- | --- | --- | --- | --- | --- | --- |
| Hamburg, Germany | 1,047 | 82.0 (6.7) | 67.6 | 33.9 (7.4) | 174.5 (9.5) | 72.1 (14.3) | 52.9 |
| Erfurt, Germany | 653 | 82.8 (6.1) | 62.8 | 33.1 (6.8) | 171.1 (8.9) | 71.2 (13. 6) | 52.2 |
| Bordeaux, France | 521 | 80.2 (6.6) | 62.8 | 31.2 (6.8) | 169.5 (8.9) | 64.6 (12.7) | 51.2 |
| Paris,  France | 615 | 85.1 (6.5) | 61.5 | 36.4 (7.1) | 169.2 (8.9) | 65.6 (12.4) | 46.8 |
| Reykjavik, Iceland | 523 | 82.9 (5.8) | 62 | 33.4 (6.9) | 173.9 (8.8) | 72.9 (13.8) | 49.7 |
| Bergen, Norway | 773 | 82.1 (5.7) | 58.9 | 33.1 (6.7) | 173.4 (9.4) | 72.7 (13.9) | 49.8 |
| Gothenburg, Sweden | 600 | 83.6 (6.3) | 58.2 | 33.5 (7.3) | 172.5 (9.6) | 70.2 (13.0) | 47.8 |
| Uppsala, Sweden | 541 | 82.2 (6.6) | 48.8 | 32.9 (7.4) | 173.6 (9.4) | 70.3 (13.8) | 49.5 |
| Basel, Switzerland | 788 | 79.4 (7.6) | 59 | 33.0 (7.1) | 171.4 (9.0) | 68.0 (13.3) | 50.5 |
| Melbourne, Australia | 552 | 78.9 (6.6) | 50 | 34.7 (6.8) | 169.4 (9.4) | 72.9 (14.2) | 51.4 |

# Supplementary Figure

Beta coefficient and 95% CI (or 95% CrI) for all risk factors across the 20 scenarios, for all methods.


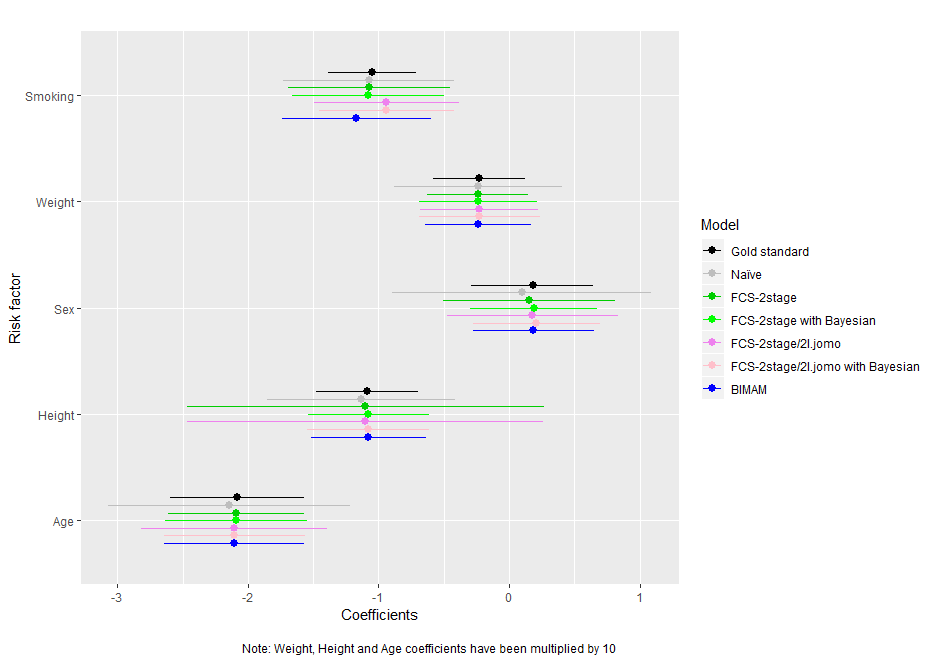


Gold standard

Naïve

FCS-2stage

FCS-2stage + Bayesian analysis model

FCS-2stage/2l.jomo

FCS-2stage/2l.jomo + Bayesian analysis model

BIMAM

**Smoking**


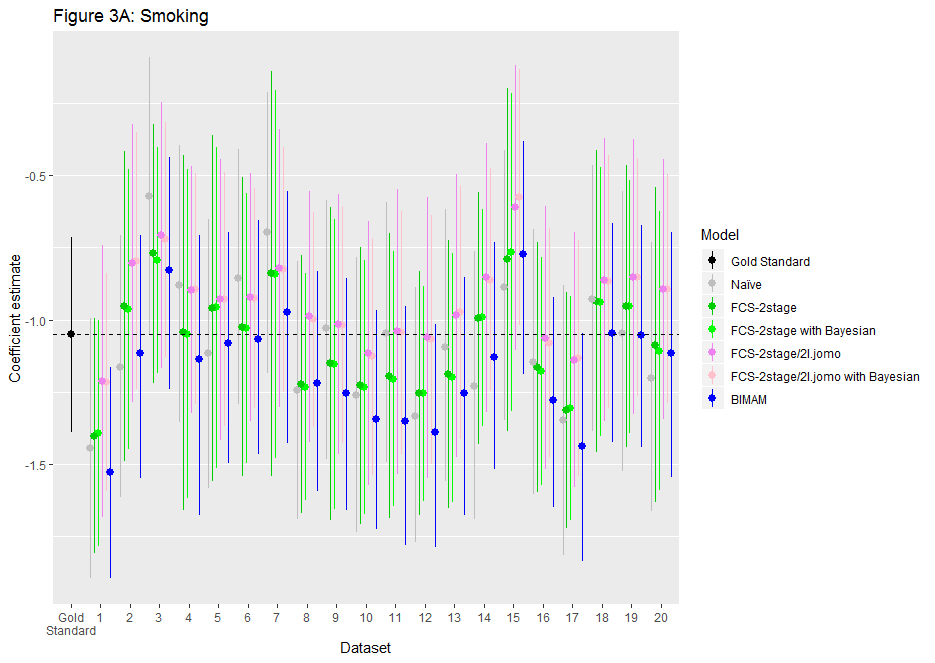


**Weight**


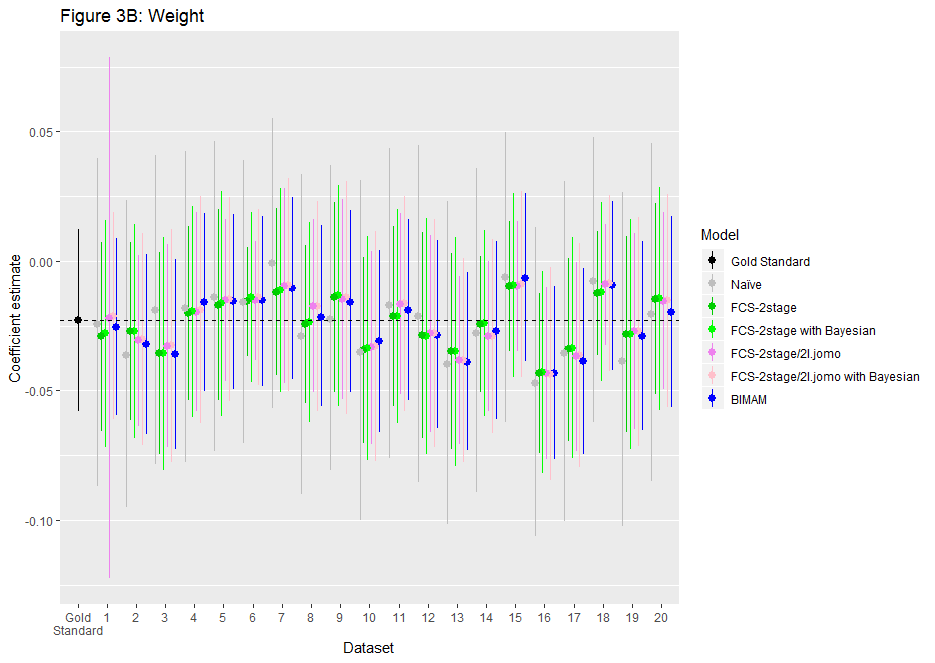


**Sex**


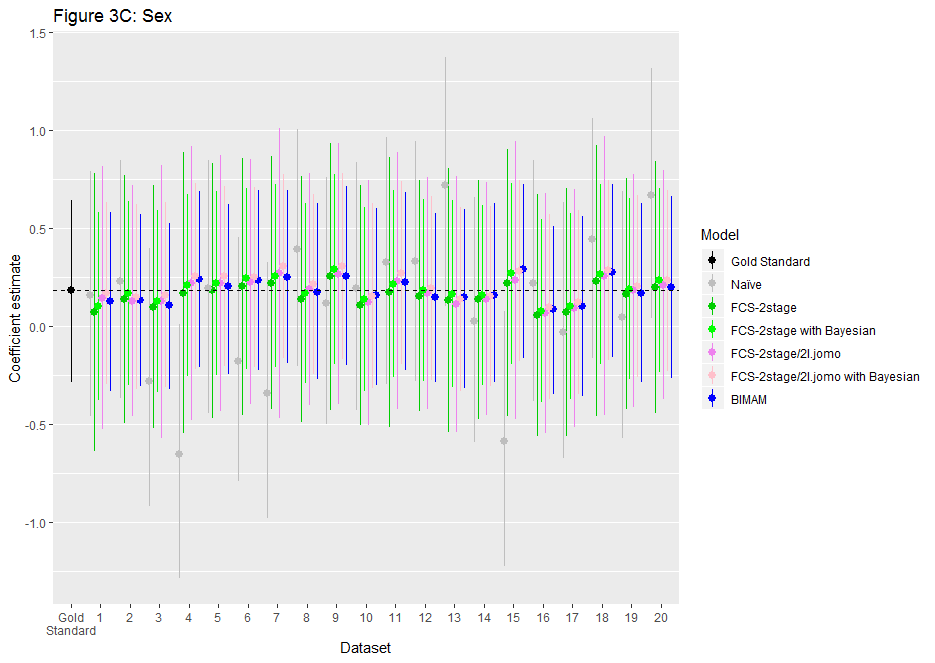


**Height**


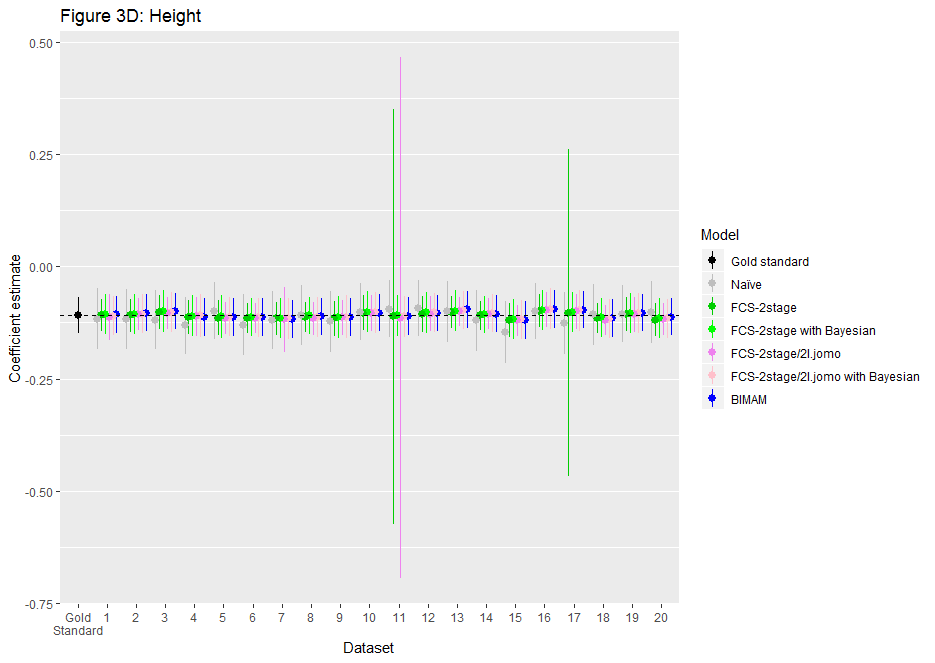


**Age**


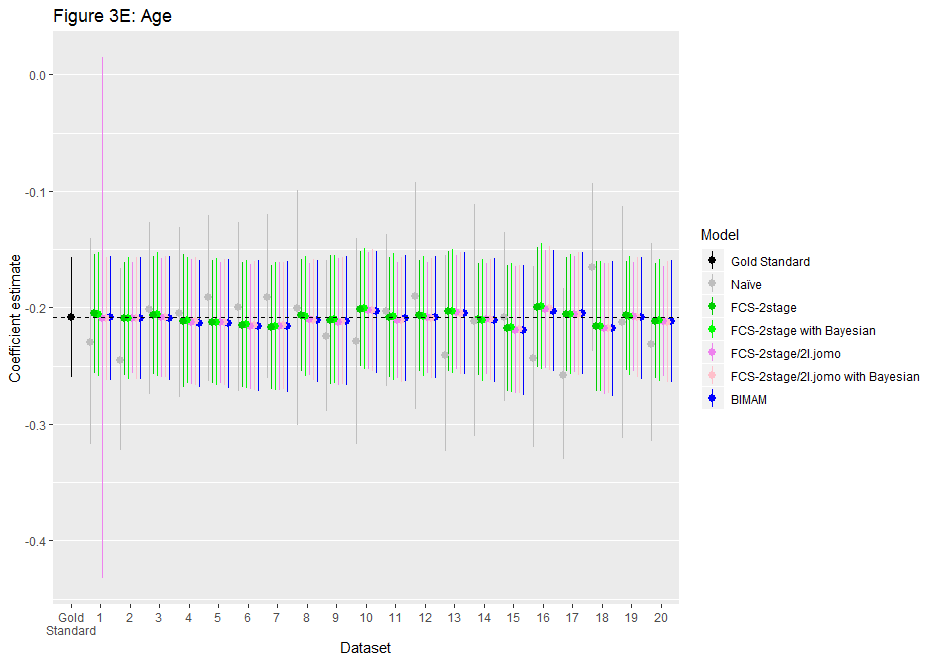


# BUGS code

The BUGS code for the Bayesian model for our illustrative example is reported below. The outcome for the analysis of interest is the FEV_1_/FVC ratio (continuous), and the covariates are sex (binary), age (continuous), height (continuous), weight (continuous) and smoking (binary).

Two variables, one binary (smoking) and one continuous (weight), are missing from some centres: 3 centres are missing both smoking and weight, one centre is missing only smoking, and one centre is missing only weight. Therefore, in total 5 centres have missing variables. The imputation model for each of the missing variables includes all other covariates, except for the other missing variable, as well as the outcome.

Random slopes for all coefficients are used in both imputation and analysis models.

The Bayesian model implemented in the tool is parameterised in the same way as described here, but the BUGS code is generalised and automatically generated based on the user’s input (see BIMAM online tool).

**#-----Imputation model--------#**

# We have two missing variables that are not independent of each other, so we create a joint distribution.

# We model the binary variable 'smoke' as a latent continuous variable with a normal distribution. This allows us to create variable 'wesmo', which is a vector of (weight, smoke), with a multivariate normal distribution.

# The variable is split into 2; 'wesmo[ ,1]' represents 'smoke' and 'wesmo[ ,2]' represents 'weight'.

for (j in 1:6613) {

# Create the imputation model to calculate weight and smoking.

# 'wesmo' is given a bivariate normal distribution

wesmo[j, 1:2] ~ dmnorm (mu.wesmo[j, 1:2], omega.wesmo[1:2, 1:2])

# To convert 'smoke' back into a binary variable based upon the latent distribution that has been given, we take the cumulative distribution function of 'mu.wesmo[j,1]' to give us a probability value between zero and one (remember, wesmo has been given a normal distribution).

# This probability is then used as the probability parameter in the Bernoulli distribution, from which a discrete binary outcome for 'smoke' is sampled.

prob.smoke[j] <- phi(mu.wesmo[j,1])

smoke[j] ~ dbern (prob.smoke[j])

# We then create an imputation model using all variables to impute values for weight and smoking.

# Imputation of weight – wesmo[,2]

mu.wesmo[j,2] <- wimp.alpha[centre[j]] + wimp.beta.age[centre[j]] * age[j] + wimp.beta.sex[centre[j]] * sex[j] + wimp.beta.fev1fvc[centre[j]] * fev1fvc[j] + wimp.beta.height[centre[j]] * height[j]

# Imputation of smoking – wesmo[,1]

mu.wesmo[j,1] <- simp.alpha[centre[j]] + simp.beta.age[centre[j]] * age[j] + simp.beta.sex[centre[j]] * sex[j] + simp.beta.fev1fvc[centre[j]] * fev1fvc[j] + simp.beta.height[centre[j]] * height[j]

# Once values have been imputed, we use the 'cut' function to prevent data from the analysis model feeding back into the imputation model.

cut_weight[j] <- cut(wesmo[j,2])

cut_smoke[j] <- cut(smoke[j])

}

**#-----Analysis model-----#**

# For each of the 6613 participants, indicated by [j], we assume that fev1fvc, a measure of lung function, is normally distributed with mean 'mu.fev1fvc' and precision 'tau.fev1fvc'

# We assume that mu.fev1fvc varies according to participant[j], whilst tau.fev1fvc is constant across all participants.

for (j in 1:6613) {

fev1fvc[j] ~ dnorm (mu.fev1fvc[j], tau.fev1fvc)

# Regression of independent variables to give outcome fev1fvc.

# Intercept 'alpha' has a random effect dependent on the participant's centre.

# 'alpha[centre[j]]' varies alpha according to the centre number of participant 'j'.

# All beta parameters are dependent upon participant only.

mu.fev1fvc[j] <- alpha[centre[j]] + beta.sex[centre[j]] * sex[j] + beta.age[centre[j]] * age[j] + beta.height[centre[j]] * height[j] + beta.weight[centre[j]] * cut_weight[j] + beta.smoke[centre[j]] * cut_smoke[j]

}

**#-----Priors-----#**

# Vague priors used.

# Priors for mean values are obtained from normal distributions with mean 0 and precision 1.0E-6.

# Priors for precision values are obtained from gamma distributions of a=1 and b=0.005.

# Because alpha and beta vary randomly according to centre, we include hyperpriors for the distribution of

# mu.variable (mean) and tau.variable (precision).

***# For the imputation model:***

# For alpha:

for (i in 1:10) {

wimp.alpha[i] ~ dnorm (mu.wimp.alpha, tau.wimp.alpha)

simp.alpha[i] ~ dnorm (mu.simp.alpha, tau.simp.alpha)

}

mu.wimp.alpha ~ dnorm (0, 1.0E-6)

tau.wimp.alpha ~ dgamma (1, 0.005)

mu.simp.alpha ~ dnorm (0, 1.0E-6)

tau.simp.alpha ~ dgamma (1, 0.005)

# For betas:

for (i in 1:10) {

wimp.beta.age[i] ~ dnorm (mu.wimp.beta.age, tau.wimp.beta.age)

wimp.beta.sex[i] ~ dnorm (mu.wimp.beta.sex, tau.wimp.beta.sex)

wimp.beta.fev1fvc[i] ~ dnorm (mu.wimp.beta.fev1fvc, tau.wimp.beta.fev1fvc)

wimp.beta.height[i] ~ dnorm (mu.wimp.beta.height, tau.wimp.beta.height)

simp.beta.age[i] ~ dnorm (mu.simp.beta.age, tau.simp.beta.age)

simp.beta.sex[i] ~ dnorm (mu.simp.beta.sex, tau.simp.beta.sex)

simp.beta.fev1fvc[i] ~ dnorm (mu.simp.beta.fev1fvc, tau.simp.beta.fev1fvc)

simp.beta.height[i] ~ dnorm (mu.simp.beta.height, tau.simp.beta.height)

}

mu.wimp.beta.age ~ dnorm (0, 1.0E-6)

mu.wimp.beta.sex ~ dnorm (0, 1.0E-6)

mu.wimp.beta.fev1fvc ~ dnorm (0, 1.0E-6)

mu.wimp.beta.height ~ dnorm (0, 1.0E-6)

tau.wimp.beta.age ~ dgamma (1, 0.005)

tau.wimp.beta.sex ~ dgamma (1, 0.005)

tau.wimp.beta.fev1fvc ~ dgamma (1, 0.005)

tau.wimp.beta.height ~ dgamma (1, 0.005)

mu.simp.beta.age ~ dnorm (0, 1.0E-6)

mu.simp.beta.sex ~ dnorm (0, 1.0E-6)

mu.simp.beta.fev1fvc ~ dnorm (0, 1.0E-6)

mu.simp.beta.height ~ dnorm (0, 1.0E-6)

tau.simp.beta.age ~ dgamma (1, 0.005)

tau.simp.beta.sex ~ dgamma (1, 0.005)

tau.simp.beta.fev1fvc ~ dgamma (1, 0.005)

tau.simp.beta.height ~ dgamma (1, 0.005)

# For the variance-covariance matrix

# (where L is an upper-triangular matrix with L[2,1]=0, and R is the correlation matrix):

omega.wesmo[1:2, 1:2] <- inverse(sigma[,])

L[1,1] <- 1

L[1,2] ~ dunif(-1,1)

R[1,2] <- L[1,1]*L[1,2]

sigma[1,1] <- pow(sd[1],2)

sigma[1,2] <- sd[1]*R[1,2]*sd[2]

sigma[2,1] <- sigma[1,2]

sigma[2,2] <- pow(sd[2],2)

sd[1] <- 1

sd[2] <- dunif(0, 10)

***# For the analysis model:***

# For alpha:

for (i in 1:10) {

alpha[i] ~ dnorm (mu.alpha, tau.alpha)

}

mu.alpha ~ dnorm (0, 1.0E-6)

tau.alpha ~ dgamma (1, 0.005)

# For betas:

for (i in 1:10) {

beta.sex[i] ~ dnorm (mu.beta.sex, tau.beta.sex)

beta.age[i] ~ dnorm (mu.beta.age, tau.beta.age)

beta.height[i] ~ dnorm (mu.beta.height, tau.beta.height)

beta.weight[i] ~ dnorm (mu.beta.weight, tau.beta.weight)

beta.smoke[i] ~ dnorm (mu.beta.smoke, tau.beta.smoke)

}

mu.beta.sex ~ dnorm (0, 1.0E-6)

mu.beta.age ~ dnorm (0, 1.0E-6)

mu.beta.height ~ dnorm (0, 1.0E-6)

mu.beta.weight ~ dnorm (0, 1.0E-6)

mu.beta.smoke ~ dnorm (0, 1.0E-6)

tau.beta.sex ~ dgamma (1, 0.005)

tau.beta.age ~ dgamma (1, 0.005)

tau.beta.height ~ dgamma (1, 0.005)

tau.beta.weight ~ dgamma (1, 0.005)

tau.beta.smoke ~ dgamma (1, 0.005)

# For the precision of the normal distribution of the outcome variable 'fev1fvc':

tau.fev1fvc ~ dgamma (1, 0.005)

}

# BIMAM online tool

BIMAM is a stand-alone user-friendly tool freely available at [www.alecstudy.org/bimam](http://www.alecstudy.org/bimam). It comes with an example dataset to illustrate its use in the online manual, which is the same ECRHS dataset used for illustration in the paper. The interface is organised with instructions provided at each step, as well as a help message next to each input field that can be seen by clicking on a question mark symbol.

After some brief information on BIMAM and instructions for use, the user is asked to upload the data as a csv or text file. The data need to be in the form of a single dataset that combines by row all the datasets the user is analysing, with a column (clustering variable) specifying which dataset each observation belongs to.

The page has five tabs for a general description with: instructions for use; citation; description of the Bayesian model; manual; contact:


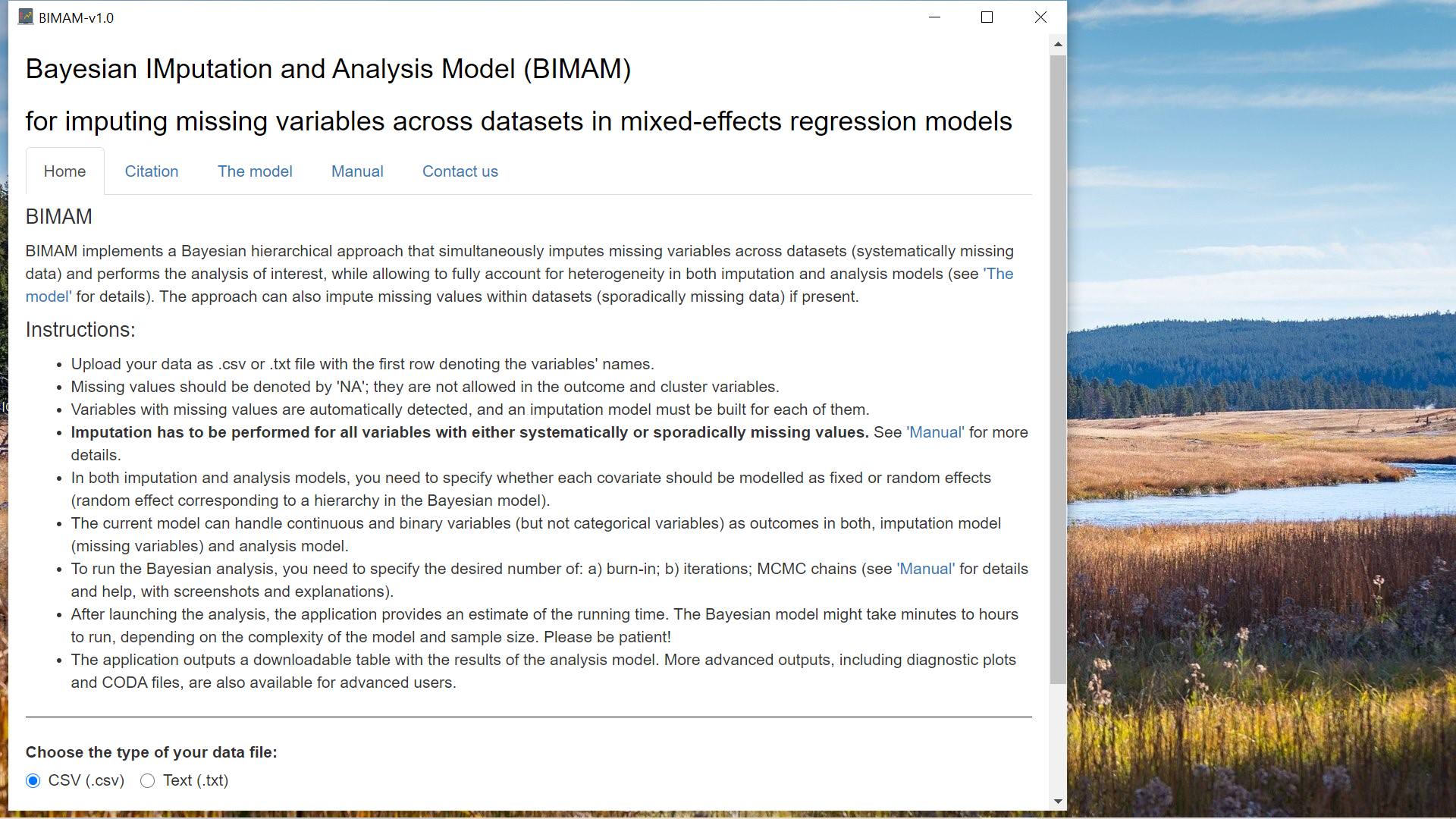


After uploading the dataset, a summary is provided with: number of variables and observations in the dataset; variables with missing data; table with descriptive statistics and number of missing values for each variable:


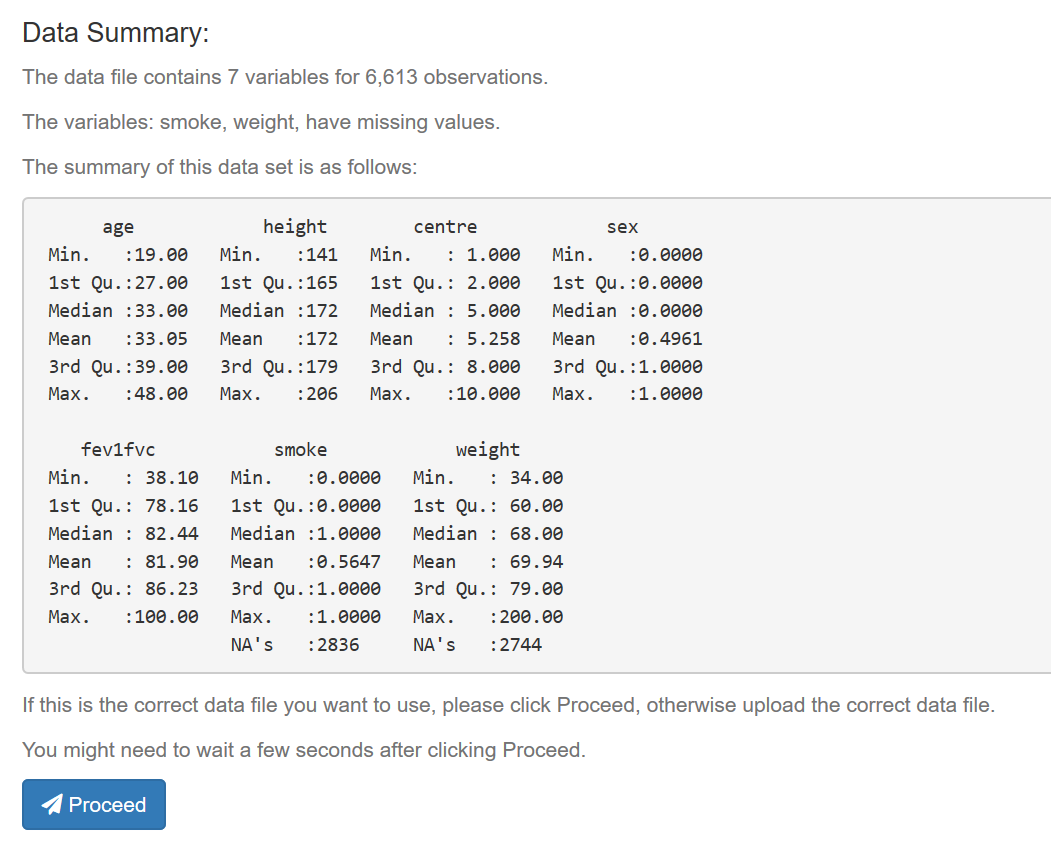


Using a drop-down list, the user is then asked to specify: a) the clustering variable (e.g. centre, study); b) outcome and covariates for both imputation and analysis models; c) for each covariate in both models, whether it is modelled as fixed or random effect:


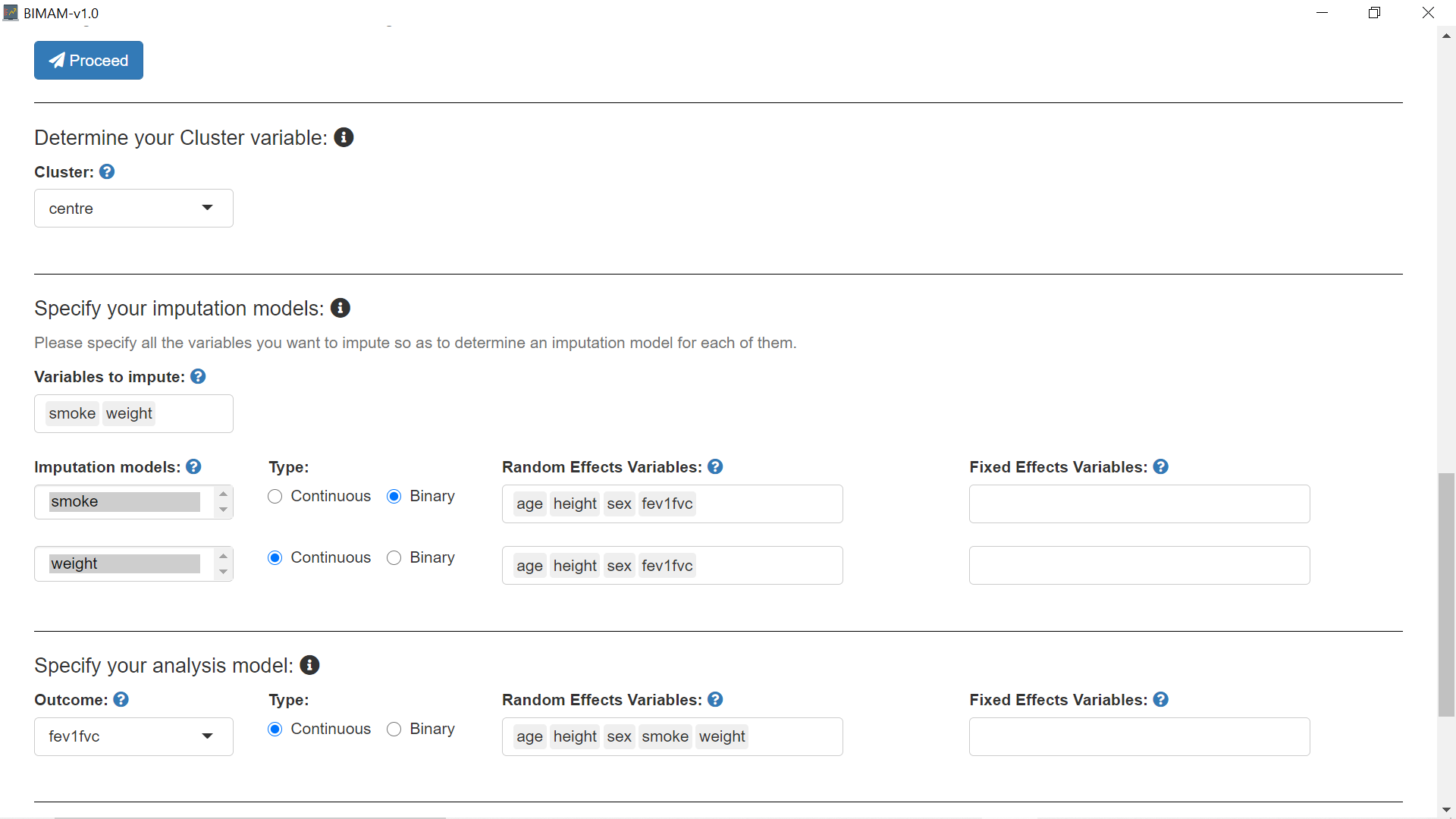


To run the MCMC analysis, the user also needs to specify: 1) length of “burn-in” (initial iterations that are discarded to avoid any influence of the initial values on the results); 2) number of “updates” (iterations used in the analysis - the larger the updates, the more accurate the results); 3) number of chains (number of separate MCMCs, used to assess model convergence). Initial values for all parameters are assigned by the tool (see Details of the Bayesian approach, p. 3). Messages to help users not familiar with Bayesian methods decide about these parameters appear by clicking on the question mark symbol, with more explanation provided in the manual:


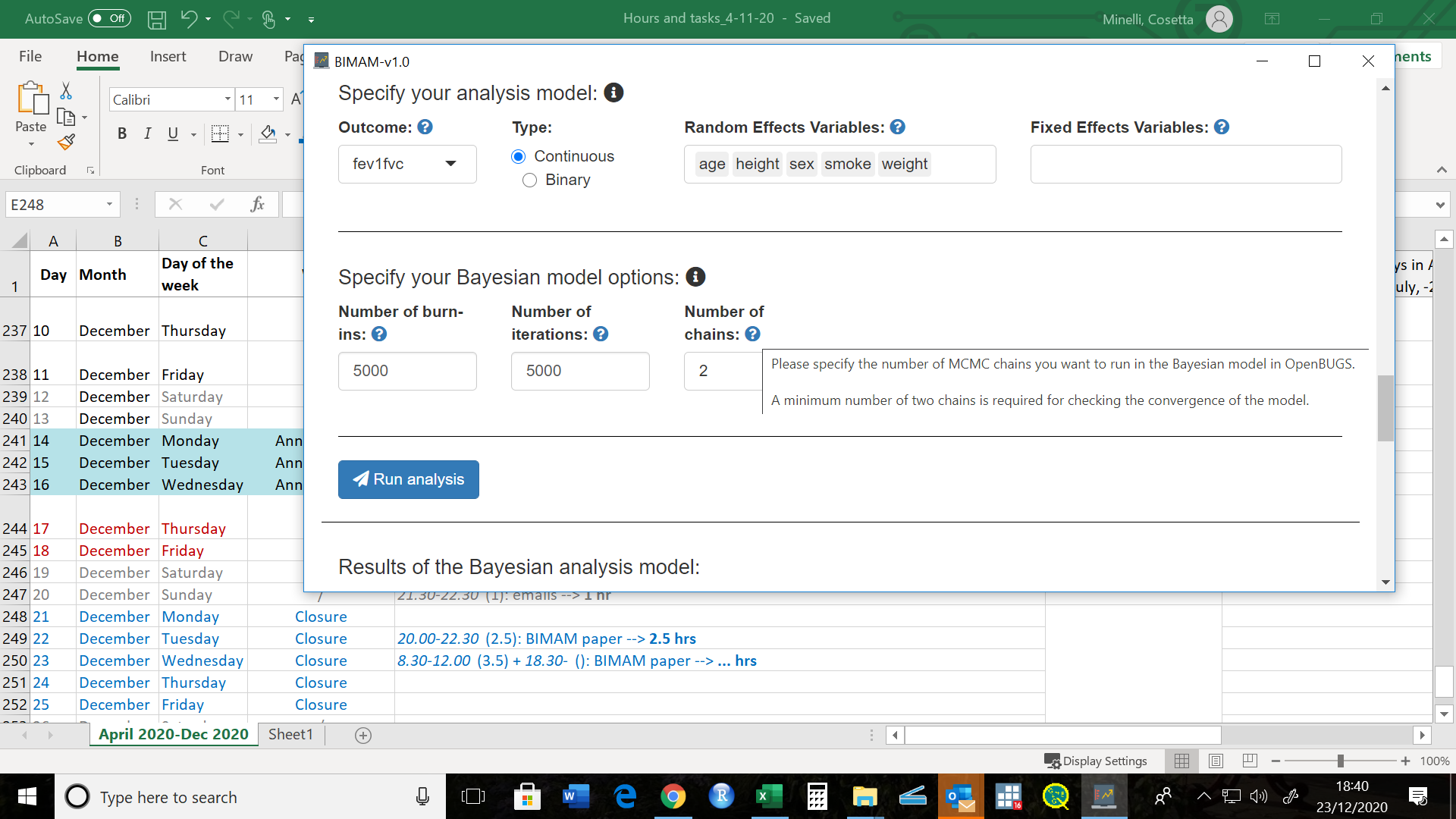


The results output presents the beta coefficients of all variables in the analysis model in a downloadable table, with posterior estimate, standard error and 95%CrI, as well as MCMC error and R-hat.


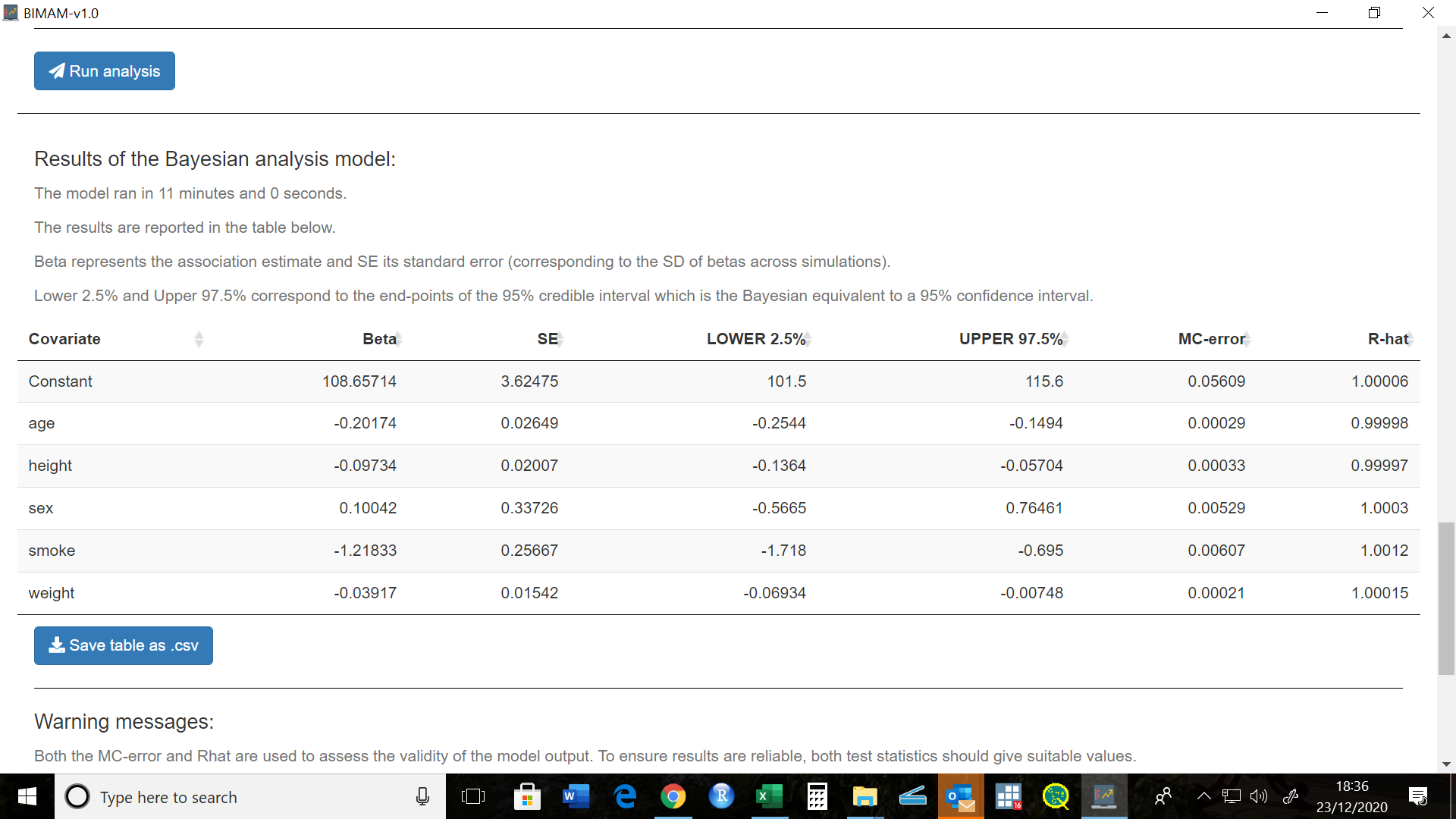


For users not familiar with Bayesian methods, meaning and implications of values of MCMC error and R-hat messages are briefly explained, with more detailed information in the manual. Warnings and recommendations are provided if: the accuracy of the results is too low 🡪 suggestion to increase number of updates if MCMC error (simulation error) > 5% of the standard error of any parameter of interest; convergence is not reached 🡪 suggestion to increase burn-in period if Gelman-Rubin statistic (R-hat) > 1.1 [5]. For advanced users, buttons are provided to view and save diagnostic plots (trace plots and density plots), and to save CODA files:


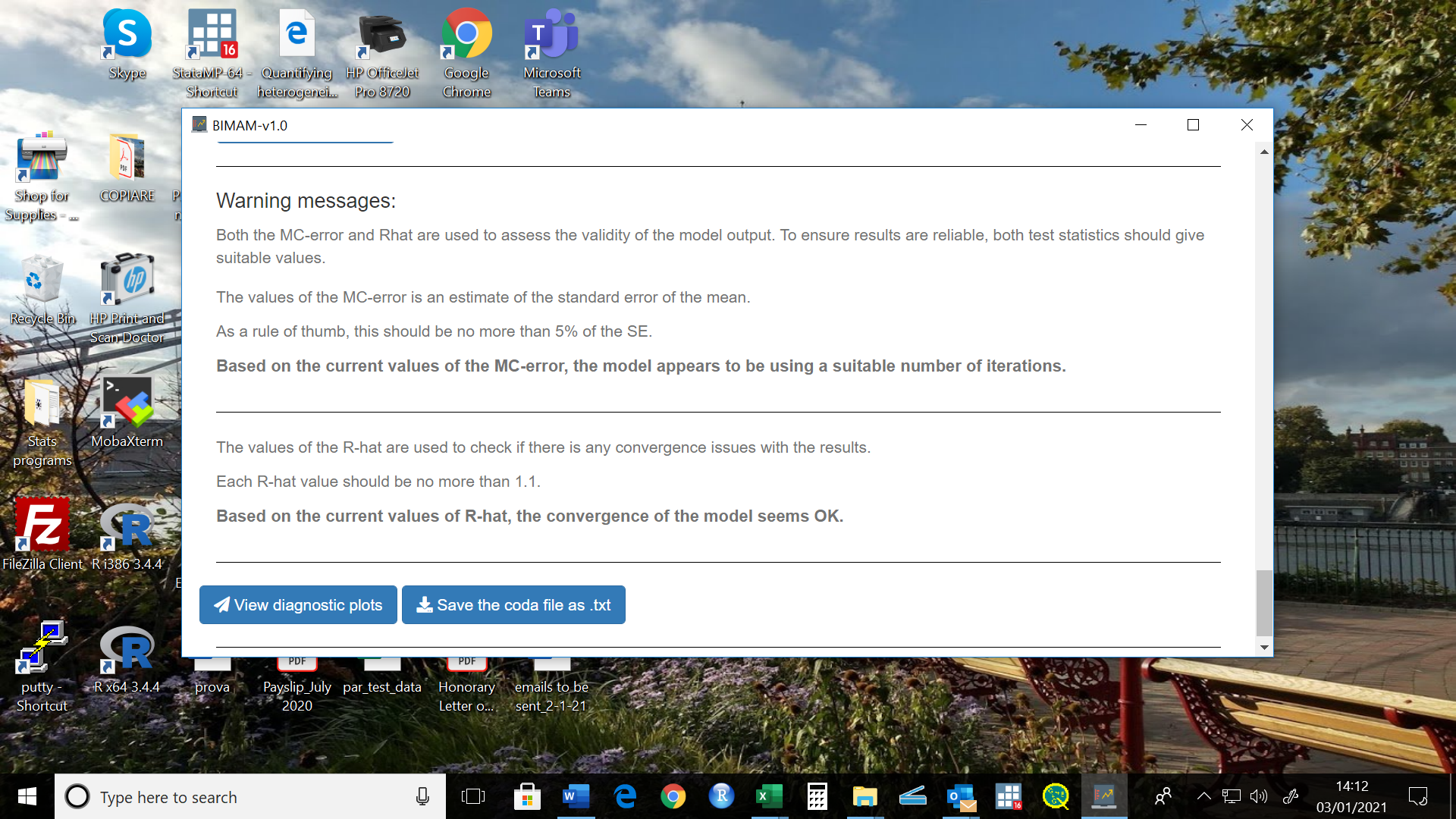


# References

1. Jackson CH, Best NG, Richardson S. Bayesian graphical models for regression on multiple data sets with different variables. Biostatistics (Oxford, England). 2009;10(2):335-51. Epub 2008/11/29. doi: 10.1093/biostatistics/kxn041. PubMed PMID: 19039032; PubMed Central PMCID: PMCPMC2648903.

2. Audigier V, White I, Jolani S, Debray T, Quartagno M, Carpenter J, et al. Multiple imputation for multilevel data with continuous and binary variables.: ArXiv e-prints <arXiv:1702.00971>; 2017.

3. Goudie RJB, Turner RM, De Angelis D, Thomas A. MultiBUGS: A Parallel Implementation of the BUGS Modelling Framework for Faster Bayesian Inference. J Stat Softw. 2020;95. PubMed PMID: 33071678.

4. Wei Y, Higgins JP. Bayesian multivariate meta-analysis with multiple outcomes. Statistics in medicine. 2013;32(17):2911-34. Epub 2013/02/07. doi: 10.1002/sim.5745. PubMed PMID: 23386217.

5. Brooks S, Gelman A. General methods for monitoring convergence of iterative simulations. Journal of Computational and Graphical Statistics. 1998;7:434–55.
